# TGF-β1 and IGF-I gene variations in type 1 diabetes microangiopathic complications

**DOI:** 10.1186/2251-6581-13-45

**Published:** 2014-04-01

**Authors:** Javad Tavakkoly Bazzaz, Mahsa M Amoli, Zahra Taheri, Bagher Larijani, Vera Pravica, Ian V Hutchinson

**Affiliations:** 1 Department of Medical Genetics, School of Medicine, Tehran University of Medical Sciences, Tehran, Iran; 2 Endocrinology and Metabolism Research Centre, Endocrinology and Metabolism Research Institute, Tehran University of Medical Sciences, Tehran, Iran; 3 School of Pharmacy, University of Southern California (USC), Los Angeles, USA

**Keywords:** T1DM, IGF-I, TGF-β1, Polymorphism

## Abstract

**Background:**

Growth factors are generally believed to have a perpetuating role in the development of diabetic complications, However there is ample of evidence of a protective or therapeutic potential for some of them. IGF-I, according to some reports, may contribute to complication development, although a protective role for IGF-I has been claimed for all late diabetic complications, making it an exception among growth factors. Transforming growth factor (TGF)-β1 as a pleiotropic cytokine is a key player in immunoregulation. Dysregulation of TGF-β1 in diabetes has been addressed as a leading event of kidney pathologies, while there is no similar pivotal role for TGF-β1 in diabetic retinopathy or neuropathy. An association study was conducted to evaluate the distinctive roles of TGF-β1 and IGF-I in T1DM microvascular complications by gene variation-based regulatory mechanisms that are operational in modulation of both *in situ* and systemic levels of the gene product.

**Methods:**

Two polymorphisms of the IGF-I gene at positions −383*C/T and −1089*C/T and two functional TGF-β1 gene polymorphisms, including codons 10 (+869*C/T) and 25 (+915*G/C) were examined in 248 British Caucasian T1DM patients and 113 healthy controls.

**Results:**

The distribution of IGF-1 gene polymorphisms did not reflect any significant association with different endpoints among the cases or different subgroups (complication triad) and controls. For TGF-β1 gene codon 25 polymorphism the low producer variant (allele C) were more frequent in cases than controls, which is compatible with the anti-inflammatory role of TGF-β1 and for codon 10 polymorphism the frequency of allele C was highest in retinopaths and, on the contrary and expectedly, nephropathy was more frequently accompanied by allele T (high producer). The frequency of allele G (high producer) of codon 25 polymorphism was slightly higher in the complication free group than in other subgroups.

**Conclusion:**

Although there were some differences in distribution of allele and genotype frequencies of TGF-β1 gene polymorphism in diabetes microvascular complications the differences were not statistically significant. Regarding IGF-1 our result firstly questions the functionality of the employed polymorphic marker and secondly may entail that the main regulator of IGF-I functionality resides elsewhere rather than the IGF-I gene itself, such as post-transcriptional regulation.

## Introduction

Growth factors are generally implicated in the development of diabetic retinopathy (DR) and diabetic nephropathy (DN), while in diabetic neuropathy (DNU) some of them (such as VEGF, IGF-I and NGF) have a protective role. This differential impact of growth factors is partly related to the discrete pathophysiological nature of these different endpoints.

The IGF-I gene is located on the long arm of chromosome 12q22-24.1 [1,2]. The encoded IGF-1 protein is a single chain polypeptide with 70 amino acids. Both IGF-I and its specific receptor (IGF-IR) have strong structural homology with pro-insulin and insulin receptor, respectively. The main mode of action of IGF-I is endocrine but, in contributing to the main pathways in the exacerbation of diabetic complications, it could function in an auto- (i.e. as an oncogene [3] or para-crine manner as well.

TGF-β1 is a homodimeric protein with a molecular weight of about 25 KDa, encoded by a gene on chromosome 19q13 [4]. There are at least seven polymorphisms in the TGF-β1 gene, but two of them have been identified as functional polymorphisms, which are associated with interindividual variation in the level of its production. These polymorphisms are located in the signal sequence at position +869*C/T, which changes codon 10 (Leu → Pro) and at position +915*G/C, which changes codon 25 (Arg → Pro) [5].

The pivotal role of TGF-β1 in development of DN (mainly due to its hypertrophic and profibrotic effects) is documented. The expression of TGF-β1 was shown to be increased 3–10 fold in the glomeruli of STZ-diabetic rats. Its elevation was both in local (glomerular) and circulating form [6]. While the local (glomerular) increase of TGF-β1 is attributable mainly to increased *in situ* expression (TGF-β mRNA) [7,8], its systemic elevation might be due to its release from platelets, which serve as a large reservoir of TGF-β. Hyperfragility of platelets in diabetes could lead to TGF-β1 release from platelets into serum [9]. The exposure of endothelial cells to increased serum TGF-β may in turn develop/precede generalised basement membrane thickening.

TGF-β1 elevation has been reported as early as 24 h following the onset of hyperglycaemia [10]. Similarly, TGF-β protein immunostaining was elevated in the kidneys of humans with diabetic nephropathy [11]. The level of intraglomerular TGF-β1 mRNA has been correlated with the staining intensity of collagen type IV in the mesangium, glomerular basement membrane, and Bowman’s capsule [8].

The elevation of all three TGF-β isoforms at the glomerular and tubolointerstitial levels along with increased extracellular matrix synthesis has been documented in different glomerular diseases, including diabetic nephropathy [12]. While the entire TGF-β axis is involved in diabetic nephropathy, TGF-β2 and TGF-β type II receptor have displayed the most prominent changes at the protein level in STZ-induced and BB-rats [13].

IGF-I serum levels usually have not been found increased in diabetic patients. Instead, there is IGF-I depletion at the systemic level, most profoundly in patients with poor glycaemic control [14]. At the tissue level, there is decreased IGF-1 availability due to diminished serum IGF-I (free and total) and elevated IGF-BP1 [15] (as an inhibitor of IGF-I) [16]. As a result of improved metabolic control, the serum level of IGF-I usually increases [17,18]. This may explain the pathophysiology of the “bush fire” phenomenon, which is transient aggravation of proliferative diabetic retinopathy (PDR) following better glycaemic control [19].

The correlation of retinal ischemia with both local IGF-I production (and also IGF-II and IGF-BPs) and angiogenesis [20] has been documented. However some studies show serum IGF-I elevation [21], while other studies reveal no connection between serum IGF-I and DR [22], some reports note its elevation at the later stage of DR (too late to be considered a cause) and some cite only its transient elevation just prior to DR commencement [23].

A considerable drop (30%) in levels of active TGF-β has been observed in PDR. In massive ocular angiogenesis due to either diabetes or non-diabetic aetiologies, the reduction of vitreous TGF-β was about ⅛ relative to control levels [9].

Recently it has been reported that in type 2 diabetics TGF-(1 gene polymorphism at codon 25 (+915*G/C) is associated with PDR (vs type 2 diabetics without PDR, p = 0.007), while the polymorphisms at positions –988C/A, −800G/A, −509C/T, and at codon 10 (+869*T/C) were not associated with PDR [24].

However, although active TGF-(1 is downregulated in retinal angiogenesis due to ischemia, it seems TGF-(1 down-regulation does not have a key role in diabetic retinopathy.

In experimental animal models, it has been documented that intravitreal injection of IGF-I in rabbits [25] and pigs [26] causes retinal neovascularisation and microangiopathy, correspondingly. Furthermore, suppression of serum IGF-I (secondary to GH down-regulation) reduced the progression of retinal neovascularisation in a mouse model of retinal ischemia [27]. The vitreous level of IGF-I in proliferative DR was 2.5 fold higher than controls [28];

Intravitreal IGF-I was significantly higher in diabetics than in controls, and the highest intravitreal level was found in insulin-treated patients with actively vascularised retinal membrane. Interestingly, among different growth factors (IGF-I, bFGF and TGF-β2), only IGF-I had a constantly higher vitreal level in all diabetic patients with proliferative DR (with regard to diabetes type or method of glycaemic control, and neovascular activity), while others appear differently in different subgroups. For instance, bFGF was higher in non-insulin treated patients and TGF-β2 was higher among those treated with insulin, but IGF-I was increased in both populations [29];

In terms of potential causes of elevated IGF-I in DR, excess secretion or release from the retina by active neovascularised membranes, contribution from systemic sources, and impaired degradation of IGF-I (IGF-BPs action) have been mentioned [29].

A considerable drop (30%) in levels of active TGF-β has been observed in PDR. In massive ocular angiogenesis due to either diabetes or non-diabetic aetiologies, the reduction of vitreous TGF-β was about ⅛ relative to control levels [9]. On the other hand, as the vitreous extract of normal individual inhibits retinal capillary growth in vitro [30], such an inhibitory property was ascribed to secretions of pericytes and smooth muscle cells [31]. However, if TGF-(is acting in a paracrine manner, its vitreous level may not reflect the local concentration.

Seemingly pericytes could regulate endothelial cell proliferation by secretion of TGF-(, and subsequently by its conversion from latent to active TGF-(. In co-culture with pericytes and smooth muscle cells, the migration of endothelial cells was inhibited by activation of a latent TGF-(−like molecule [32] and likewise co-culture with pericytes resulted in therelease of active TGF-(, which is a likely inhibitor of angiogenesis by endothelial cells in the eye [33]. These observations are consistent with pericyte loss as a prodromal event in endothelial cell proliferation in DR.

Recently it has been reported that in type 2 diabetics TGF-(1 gene polymorphism at codon 25 (+915*G/C) is associated with PDR (vs type 2 diabetics without PDR, p = 0.007), while the polymorphisms at positions –988C/A, −800G/A, −509C/T, and at codon 10 (+869*T/C) were not associated with PDR [24].

However, although active TGF-(1 is downregulated in retinal angiogenesis due to ischemia, it seems TGF-(1 down-regulation does not have a key role in diabetic retinopathy.

With regard to diabetic neuropathy IGF-I decrease growth factor synthesis by target organs, disrupt retrograde transport of growth factors to the neuronal cell body, effect of signal transduction mechanisms of growth factors in neurons, alteration of the ability of neurons or Schwann cells to produce growth factors required for normal cell maintenance [34]. a) IGF-I (and IGF-II) mRNA was significantly decreased in peripheral nerves, early after development of diabetes in rats in comparison to non-diabetic rats.

It has been supposed that IGF-I (and IGF-II) has a neurotrophic effect and promotes nerve regeneration as well. The rate of nerve regeneration is reduced by antibodies to both IGF-I and IGF-II [35]. Neurite (axon and dendrite) sprouting in muscle is intensified by IGF-I and IGF-II [36]. A significant decrease in systemic IGF-I (together with elevated IGFBP-1) has been reported in patients with severe neuropathic complications [37]. However, it is not known that how IGFs could provoke nerve regeneration.

NGF, IGF-I and ciliary neurotrophic factor (CNTF) are crucial players in the normal growth, maintenance and regeneration of the peripheral nervous system, but there is no such role for TGF-(1 in diabetic neuropathy. However, the lack of TGF-(1 up-regulation has been proposed as the reason for impaired healing in diabetic foot ulcers [38].

Given that IGF-I and TGF-β1 have established role in the diabetic complication triad, we sought to determine whether their genetic variations encode the genetic predisposition to diabetic complications. Two polymorphisms of the IGF-I gene at positions −383*C/T and −1089*C/T were used as markers in a candidate gene association study. Also the association of two SNPs of TGF-β1 gene at positions +869*T/C and +915*G/C with susceptibility to T1DM and the development of late complications of diabetes was examined.

### Patients and controls

As an out-patient clinic in the North West region of the UK, about 5000 T1DM registered patients are regularly attending to the Manchester Diabetes Centre. In the present cross-sectional study, in total 248 unrelated British Caucasian attendees with T1DM were randomly selected during 1999–2002 as the patient group. The ethical approval was obtained from the Manchester Royal Infirmary. Written informed consent was obtained from all patients attending the study.

All patients fulfilled the relevant criteria for related diagnosis as are detailed later. To be on the safe side, patients who had diabetes less than 3 years were excluded from analysis.

#### Type 1 diabetes

Diabetes was diagnosed according to the criteria, which was suggested by an expert committee in 1997 (report of the expert committee on the diagnosis and classification of diabetes Mellitus, [39]). The diabetic patients included in the present study fulfilled at least one of the triple criteria recommended by the expert committee, detailed as follows:

a) Symptoms of hyperglycaemia (polyuria, polydipisia, unexplained weight loss) plus random plasma glucose > 200 mg/dL (11.1 mmol/L). Random is defined as any time of day without regard to time since last meal.

b) Fasting plasma glucose (FPG) > 126 mg/dl (7.0 mmol/L). Fasting is defined as no caloric intake for at least 8 hours.

c) 2-hour plasma glucose (PG) > 200 mg/dl (11.1 mmol/L) during an oral glucose tolerance test (OGTT). The test should be performed as described by the World Health Organization (WHO), using a glucose load containing the equivalent of 75-g anhydrous glucose dissolved in water.

Diabetes was regarded as T1DM if it was diagnosed before age of 30 years and accompanied with acute onset and treatment with insulin began within the first year of diagnosis and continued thereafter.

#### Diabetic retinopathy (DR)

The back of the eye was examined by fundoscopy (after pupillary dilatation) and when more than five dots or blots per eye, hard or soft exudates or new vessels were evident the diagnosis of retinopathy was applied. Patients who had a history of laser treatment were also diagnosed as retinopathy.

#### Diabetic nephropathy (DN)

The elevation of AER (> 300 mg-2 g/day) at least on two of three occasions and/or 3 positive Albustix over the past 12 months were evident to mark patients as nephropath, while a urinary tract infection (UTI) was ruled out already.

#### Diabetic neuropathy (DNU)

Neurothesiometer -a clinical electromagnetic vibrating device- was applied for screening of peripheral diabetic neuropathy, which quantifies the vibration sensitivity through the measurement of vibratory perception threshold (VPT), while the patient’s eye is closed and the probe of neurothesiometer is placed on the hallux of the toe. An average of 3 readings were taken. DNU was diagnosed when VPT was more than 25 volts (vibration threshold above 25 volts indicates a high risk of ulceration). The symptoms of sensory and/or motor neuropathy were looked for, like parasthesia, numbness, tingling, nocturnal rest ache, all in the absence of peripheral vascular disease as non-specific (non-diabetic) underlying cause. The excluding of peripheral vascular disease was approved by palpable pulses and measuring of ankle brachial pressure index.

## Materials and methods

ARMS-PCR was carried out to genotype healthy controls and patients for TGF-β1 gene polymorphisms at codons 10 (+869*T/C) and 25 (+915*G/C) using as previously described [40]. Also the ARMS-PCR method was developed for genotyping of IGF-I (−383*C/T and −1089*C/T) polymorphisms ([41]; unpublished data). A non-polymorphic fragment (425 bp in length) of the human growth hormone (HGH) gene also was chosen as a target for internal control primers (Table 1).

**Table 1:** The primers sequences for genotyping of IGF-1 and TGF–β1 polymorphisms

**Internal control primers (Human Growth Hormone)**		**PCR product size**
HGH1 5′- GCCTTCCCAACCATTCCCTTA-3′		425 bp
HGH2 5′- CAAGGATTTCTGTTGTGTTTC-3′		
**Gene specific primers**		
IGF-I (−383*C/T)	5′-GTGACAGGCAGCCTAGTAGA-3′	189 bp
	5′-TCCCAGTTGCCAAGTGAGG-3′	
	5′-GTCCCAGTTGCCAAGTGAGA-3′	
IGF-I (−1089*C/T)	5′-CACTTGCCTTTGCCATTGAG-3′	244 bp
	5′-AGTCCCCTGAGAGTCATGC-3′	
	5′-CAGTCCCCTGAGAGTCATGT-3′	
TGF–β1 (Codon 10)	5′- TCCGTGGGATACTGAGACAC- 3′	241 bp
	5′- GCAGCGGTAGCAGCAGCG-3′	
	5′- AGCAGCGGTAGCAGCAGCA-3′	
TGF–β1 (Codon 25)	5′- GGCTCCGGTTCTGCACTC-3′	233 bp
	5′- GTGCTGACGCCTGGCCG-3′	
	5′- GTGCTGACGCCTGGCCC-3′	

A master mix solution was used for DNA amplification. This master mix includes 22% of Ready Load Reaction Buffer (AB Technology, UK), 22% of 200 μM dNTPs (AB Technology, UK), 13% of 1.5 mM magnesium chloride (AB Technology, UK), 31% of 60% (W/V) sucrose, 11% internal control primer (1 μM each), and 1.1% of Thermoprime DNA Polymerase (AB Technology, UK). For each sample, 1.5 μl of DNA was added to 15 μl of the master mix solution and then 5 μl of specific primer mix was aliquoted to 5 μl of master mix solution, which already contains DNA as well. After that, this 10 μl reaction was amplified on a PTC-100 PCR thermal cycler (MJ Research, Inc), where the cycles was programmed as follows: 1 minute at 96°C followed by 10 cycles of 15 seconds at 95°C, 50 seconds at 65°C, 40 seconds at 72°C followed by 20 cycles of 20 seconds at 95°C, 50 seconds at 59°C and 50 seconds at 72°C.

In gel electrophoresis, according to the presence or absence of amplified targeted sequence, the type of alleles (genotype) are identified. The amplified products were visualized in a 2% agarose gel against 200 bp ladders and stained with 5 μl (0.5 mg/ml) of ethidium bromide.

### Statistical analysis

Strength of association between different groups and alleles or genotypes of polymorphisms were estimated using odds ratios (OR) and 95% confidence intervals (CI). Levels of significance were determined using contingency tables by either Chi-square or Fisher exact analysis using the STATA (v8) software (STATA Software, College Station, Texas, USA).

## Results

**Figure 1:**
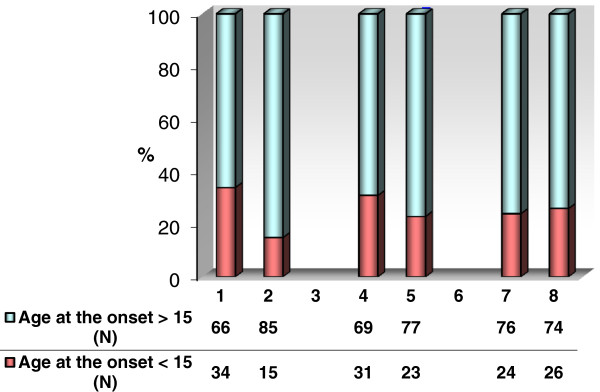
The comparative illustration of triad of microvascular diabetic complications according to the age at the onset of diabetes (under or over 15 years).

Figure 1 describes the association between age at onset of diabetes and development of complications in our patients. Among triad of diabetic complications only in DR the impact of age at the onset was statistically determinant (p = 0.0008) for the development of complications. The impact of the age at the onset was insignificant for the development of both DN (p = 0.2) and DNU (p = 0.7).

The male/female ratio was 113/89 and mean age of patients was 41.8 ± 13.1 and mean duration of diabetes was 18.3 ± 11.7 while mean duration of diabetes among complication free patients was 10.9 ± 7.8.

The distribution of IGF-I gene polymorphisms at positions −383*C/T and −1089*C/T were examined by ARMS-PCR in 248 diabetic subjects and 113 healthy controls (Tables 2 and 3). The distribution of these polymorphisms did not associate with any significant differences among cases (group/subgroups) and controls.

**Table 2:** Distribution of genotype and allele frequencies of the IGF-I −383*C/T polymorphism in healthy controls (C), T1DM subjects (P), diabetic retinopaths (DR), nephropaths (DN), neuropaths (DNU) and complication free (CF) group*

IGF-I −383*C/T	**C n (%)**	**P n (%)**	**DR n (%)**	**DN n (%)**	**DNU n (%)**	**CF n (%)**
Genotype						
CC	89 (91.0)	223 (90.0)	121 (89.6)	78 (90.7)	78 (95.0)	90 (92.0)
CT	8 (8.0)	23 (9.2)	12 (8.9)	7 (8.1)	4 (5.0)	7 (7.0)
TT	1 (1.0)	2 (0.8)	2 (1.5)	1 (1.2)	0 (0.0)	1 (1.0)
Allele						
C	186 (95.0)	469 (94.6)	254 (94.0)	163 (94.8)	160 (95.0)	187 (95.4)
T	10 (5.0)	27 (5.4)	16 (6.0)	9 (5.2)	4 (5.0)	9 (4.6)

*No significant difference was evident between any groups/subgroups.

**Table 3:** Distribution of genotype and allele frequencies of the IGF-I −1089*C/T polymorphism in healthy controls (C), T1DM subjects (P), diabetic retinopaths (DR), nephropaths (DN), neuropaths (DNU) and complication free (CF) group*

IGF-I −1089*C/T	**C n (%)**	**P n (%)**	**DR n (%)**	**DN n (%)**	**DNU n (%)**	**CF n (%)**
**Genotype**						
**CC**	8 (7.0)	13 (5.0)	6 (4.4)	5 (5.8)	4 (4.9)	4 (4.0)
**CT**	50 (44.3)	99 (40.0)	49 (36.3)	34 (39.5)	35 (42.7)	40 (41.0)
**TT**	55 (48.7)	136 (55.0)	80 (59.3)	47 (54.7)	43 (52.4)	54 (55.0)
**Allele**						
**C**	66 (29.0)	125 (25.0)	61 (22.6)	44 (25.6)	43 (26.2)	48 (24.5)
**T**	160 (71.0)	371 (75.0)	209 (77.4)	128 (74.4)	121 (73.8)	148 (75.5)

*No significant difference was evident between any groups/subgroups.

The distributions of TGF-β1 gene polymorphisms were evaluated in 248 diabetic subjects and 119 healthy controls. The distribution of these polymorphisms was not significantly different in cases (group/subgroups) and controls (Tables 4 and 5).

**Table 4:** Distribution of genotype and allele frequencies of the TGF-β1 at codon 10*C/T polymorphism in healthy controls (C), T1DM subjects (P), diabetic retinopaths (DR), nephropaths (DN), neuropaths (DNU) and complication free (CF) group*

TGF-β1 codon 10*C/T	**C n (%)**	**P n (%)**	**DR n (%)**	**DN n (%)**	**DNU n (%)**	**CF n (%)**
**Genotype**						
**CC**	15 (12.5)	39 (15.7)	23 (17)	12 (14)	13 (15.85)	19 (19.39)
**CT**	57 (48.0)	126 (50.8)	69 (51.1)	43 (50)	40 (48.78)	47 (47.96)
**TT**	47 (39.5)	83 (33.5)	43 (31.9)	31 (36)	29 (35.37)	32 (32.65)
**Allele**						
**C**	87 (36.5)	204 (41.1)	115 (42.6)	67 (39)	66 (40.24)	85 (43.37)
**T**	151 (63.5)	292 (58.9)	155 (57.4)	105 (61)	98 (59.76)	111 (56.63)

*No significant difference was evident between any groups/subgroups.

**Table 5:** Distribution of genotype and allele frequencies of the TGF-β1 at codon 25*G/C polymorphism in healthy controls (C), T1DM subjects (P), diabetic retinopaths (DR), nephropaths (DN), neuropaths (DNU) and complication free (CF) group*

TGF-β1 codon 25* G/C	**C n (%)**	**P n (%)**	**DR n (%)**	**DN n (%)**	**DNU n (%)**	**CF n (%)**
**Genotype**						
**GG**	97 (81.5)	201 (81)	104 (77)	68 (79)	65 (79.3)	83 (84.7)
**GC**	21 (17.6)	44 (17.8)	28 (20.8)	17 (19.8)	16 (19.5)	15 (15.3)
**CC**	1 (0.9)	3 (1.2)	3 (2.2)	1 (1.2)	1 (1.2)	0 (0.00)
**Allele**						
**G**	215 (90.3)	446 (90)	236 (87.4)	153 (89)	146 (89)	181 (92.3)
**C**	23 (9.7)	50 (10)	34 (12.6)	19 (11)	18 (11)	15 (7.7)

*No significant difference was evident between any groups/subgroups.

## Discussions

In present study there was no significant association in either TGF-β1 allele or genotype frequency across the different groups/subgroups with multiple comparisons, but comparatively there were some points for consideration.

In polymorphism at codon 10*C/T the frequency of allele C (low producer variant) was constantly increased among patients, while a meaningful variation of its frequency was also evident among different subgroups. The constant increase of the allele C in patients and different subgroups can be explained by the immunoregulatory or anti-inflammatory function of TGF-β1, that is probably attenuated in allele C carriers. The highest frequency of allele C was found in diabetic subjects without the triad of complications (complication free group), but it was not meaningful as, for instance, patients with DR were frequently the carrier of allele C, too.

Among different complications, the highest frequency of allele C was present in subjects with DR, which may feebly suggest again that low level of TGF-β1 is favored in DR. Among cases the highest frequency of allele T (high producer variant) was present in the subgroup of diabetic nephropathy, which can be explained by the most prominent role of TGF-β1 in development of DN, but it was still lower than healthy controls, which is explicable by the pre-selection of allele C by the preceding disease (diabetes). However, when diabetic subjects were compared with each other according to presence or absence of DN, the allele distribution showed no significant difference (p = 0.47).

With respect to the polymorphism at codon 25 (+915*G/C), there was no significant association between the polymorphic alleles/genotypes with different groups and sub-groups, as well. In accordance with TGF-β1 codon 10 polymorphism, the low producer variant (allele C) was more frequent in cases than controls, which is compatible with the anti-inflammatory role of TGF-β1. With regard to the frequency of allele G (high producer variant), while the patients as a whole and also patients with different complications reflected a lower frequency of this allele, the complication free subgroup possessed a higher frequency than controls. While this feature is explainable by a protective role of the high producer allele (G) against development of diabetic complications, it contrasts with the distribution of codon 10 variants, where the low producer variant (allele C) frequency was highest in the complication free group. Nevertheless, this conflicting substantiation may question seriously the soundness of dividing criteria applied for patients’ stratification, as it was based on negative findings (absense of complications) rather than positive findings to label the complication free patients as a homogeneous group.

The highest frequency of allele C (the low producer variant) was evident in subjects with diabetic retinopathy that is compatible with functional property of TGF-β1 as an anti-proliferative mediator. It is also consistent with the data of TGF-β1 codon 10 polymorphism in DR.

However, since the main intent for analysis of TGF-β1 gene polymorphism in the present study was to explore its impact on development of DN, it was expected to observe an increase in frequency of high producer variants (allele T and G, relative to polymorphisms at codon 10 and 25, respectively) in DN. Though a number of studies corroborated that expectation, but it was not evident in this study. As among the triad of diabetic complications, only DN can lead to death (since kidney is a vital organ), non-association findings may be false, secondary to the “survivor” (selective survival) effect. It entails that in cross sectional studies, like the present study, some fraction of the risky genotypes (associated with mortal traits) are previously excluded from the study population by death, and subsequent under-representation of such risky genotypes among cases could potentially lead to a false negative association.

In accordance with our data similar negative results have been reported in advanced diabetic nephropathy, while a higher number of alleles (3 in coding region and 2 in promoter region) in TGF-β1 gene was examined [42].

Since the local expression/activation of TGF-β1 would contribute to the proliferative phase of DR, the increased frequency of high producer variants of TGF-β1 in this study also are explicable and expected in DR, as has been documented recently [24].

The “no association” result between TGF-β1 gene polymorphisms and total IDDM group may imply that the proposed role of TGF-β1 in the induction and development of IDDM might be distal and non-decisive, for example, in comparison to other candidate cytokines, like TNF-α or IFN-γ.

Given the tissue damage that occurs frequently in diabetes [43], an elevation of IGF-I is increasingly required [43], in contrast IGF-I levels are progressively decreasing in diabetes, preceding the formation of late complications. Consequently, at the tissue level, particularly in the kidneys, eyes and neurons, there is a deprivation of IGF-I. This rationale also is supported by previous findings documenting a protecting role for IGF-I in DNU due to its tissue regenerating [37] and anti-apoptotic (in glucose-induced apoptosis) properties [44]. Ishii in 1986–1987 had presented the same speculation with regard to the significant suppressive role of IGF-I and II in DNU (reviewed in [34]). The “IGF-I deficiency” theory can be viewed as an upgrade of the “GH hyper-secretion” hypothesis [45], reducing the emphasis on the role of GH, and acknowledging that the GH-IGF axis is deranged in diabetes.

In contrast to such strong evidence and facts implying a decisive role for IGF-I in diabetic complications, we found no association between IGF-I gene polymorphisms and the development of T1DM or its late microangiopathic complications. Such non-association results cannot necessarily rule out or discount the involvement of IGF-I in the development of either diabetes or late complications. It can only suggest that the pair of IGF-I polymorphisms (but not other potential polymorphisms and more notably the IGF-I gene/molecule itself) do not influence the development of those pathologies in the studied population.

With regard to remarkable alteration of IGF-I levels particularly in diabetic complications (detailed above) it strongly suggests other regulatory mechanisms i.e. post transcriptional rather than gene polymorphism based, may be involved. Even more, by the same concentration/expression different impact/outcome can result from IGF-I through the modulating role of IGF-I binding proteins and IGF-I receptors.

It also can be envisaged that due to the essential role of IGF-I in physiological homeostasis, mainly via its metabolic properties, the expression of IGF-I as a vital molecule (similar to house keeping genes products) is not determined by a quantative (rheostat-like) switching mechanism and thus does not accommodate quantative changes at the transcriptional level (i.e. due to the impact of any polymorphism) but instead it may comply with on/off binary switches, transcription rate being constant during the “on” phase [46].

In terms of the statistical view, some points can be underlined. Firstly, when the frequency of a variant allele is very low (as the allele T of the −383*C/T polymorphism), it generally weakens the power of that polymorphism as a marker. Such low frequency can be due to its non-functionality implying that it has been unaffected by and excluded from selective forces and thus cannot be accounted for as a phenotype modifier and therefore an efficient genetic marker.

However, if that polymorphism is functional, both advantageous and disadvantageous effects are feasibly explicable in different ways. If it is advantageous it might be a newly arisen mutation that has not had enough time to spread across population and be more frequent secondary to selective forces. If it is disadvantageous, the allele may be in the process of extinction. Therefore, it is too late now to evaluate its effects while it is near to entire elimination. In both these circumstances, even when it is functional, that polymorphism again is not a reliable marker since it was not actually present (too early/too late exposure) in the majority of the study population (both cases and patients) to affect the progression of diabetic complications.

One of the main limitations in our study was small sample size. Low frequency polymorphic alleles, even in the case of their relevance and connection with the disease, require a massive number of cases and controls to identify reliably its impact on the disease. For example, with the number of patients with different complications and of controls in the present study in the case of IGF-I −383*C/T to achieve significance at the 0.05 level, power calculations show it is only possible to discover its impact if a very significant relative risk is expected.

However, the role of IGF-I in the development of diabetic complications is almost certain and our findings only indicate that that influence of IGF-I might not be dictated by the examined SNPs. If other variations of IGF-I is detected, the next step is to pursue the task of this study by using them as new gene markers.

## Conclusion

In summary, the discrepancies of finding in this study compared to previous studies may reflect the dynamic nature and diverse outcome of biological phenomenon that are regulated by different mechanisms at different levels.

These results may lead us erroneously to consider a deterministic link between genotypes and phenotypes in complex traits.

## Competing interests

The authors declare that they have no competing interests.

## Authors’ contributions

JTB conceived the study, collected the samples, carried the molecular genetic study and drafted the manuscript, MMA drafted the manuscript, ZT drafted the manuscript, BL gave valuable advice which helped in drafting the manuscript, VP conceived the study, IV was principle investigator and conceived the study. All authors read and approved the final manuscript.

## References

[B1] Brissenden JE Ullrich A Francke U Human chromosomal mapping of genes for insulin-like growth factors I and II and epidermal growth factor Nature 1984 310 781 784 10.1038/310781a0 6382023

[B2] Rotwein P Structure, evolution, expression and regulation of insulin-like growth factors I and II Growth Factors 1991 5 3 18 10.3109/08977199109000267 1772660

[B3] Baserga R The IGF-I receptor in cancer research Exp Cell Res 1999 253 1 6 10.1006/excr.1999.4667 10579905

[B4] Fujii D Brissenden JE Derynck R Francke U Transforming growth factor beta gene maps to human chromosome 19 long arm and to mouse chromosome 7 Somat Cell Mol Genet 1986 12 281 288 10.1007/BF01570787 3459257

[B5] Awad MR El-Gamel A Hasleton P Turner DM Sinnott PJ Hutchinson IV Genotypic variation in the transforming growth factor-beta1 gene: association with transforming growth factor-beta1 production, fibrotic lung disease, and graft fibrosis after lung transplantation Transplantation 1998 66 1014 1020 10.1097/00007890-199810270-00009 9808485

[B6] Bollineni JS Reddi AS Transforming growth factor-beta 1 enhances glomerular collagen synthesis in diabetic rats Diabetes 1993 42 1673 1677 10.2337/diab.42.11.1673 8405711

[B7] Sharma K Ziyadeh FN Renal hypertrophy is associated with up-regulation of TGF-beta 1 gene expression in diabetic BB rat and NOD mouse Am J Physiol 1994 267 F1094-01 7810696 10.1152/ajprenal.1994.267.6.F1094

[B8] Iwano M Kubo A Nishino T Sato H Nishioka H Akai Y Kurioka H Fujii Y Kanauchi M Shiiki H Dohi K Quantification of glomerular TGF-beta 1 mRNA in patients with diabetes mellitus Kidney Int 1996 49 1120 1126 10.1038/ki.1996.162 8691733

[B9] Pfeiffer A Schatz H Diabetic microvascular complications and growth factors Exp Clin Endocrinol Diabetes 1995 103 7 14 10.1055/s-0029-1211323 7621107

[B10] Shankland SJ Scholey JW Ly H Thai K Expression of transforming growth factor-beta 1 during diabetic renal hypertrophy Kidney Int 1994 46 430 442 10.1038/ki.1994.291 7967355

[B11] Yamamoto T Nakamura T Noble NA Ruoslahti E Border WA Expression of transforming growth factor beta is elevated in human and experimental diabetic nephropathy Proc Natl Acad Sci U S A 1993 90 1814 1818 10.1073/pnas.90.5.1814 7680480 PMC45970

[B12] Yamamoto T Noble NA Cohen AH Nast CC Hishida A Gold LI Border WA Expression of transforming growth factor-beta isoforms in human glomerular diseases Kidney Int 1996 49 461 469 10.1038/ki.1996.65 8821830

[B13] Hill C Flyvbjerg A Gronbaek H Petrik J Hill DJ Thomas CR Sheppard MC Logan A The renal expression of transforming growth factor-beta isoforms and their receptors in acute and chronic experimental diabetes in rats Endocrinology 2000 141 1196 1208 10698197 10.1210/endo.141.3.7359

[B14] Amiel SA Sherwin RS Hintz RL Gertner JM Press CM Tamborlane WV Effect of diabetes and its controL on insulin-like growth factors in the young subject with type I diabetes Diabetes 1984 33 1175 1179 10.2337/diab.33.12.1175 6389234

[B15] Janssen JA Lamberts SW Circulating IGF-I and its protective role in the pathogenesis of diabetic angiopathy Clin Endocrinol (Oxf) 2000 52 1 9 10.1046/j.1365-2265.2000.00922.x 10651746

[B16] Taylor AM Dunger DB Preece MA Holly JM Smith CP Wass JA Patel S Tate VE The growth hormone independent insulin-like growth factor-I binding protein BP-28 is associated with serum insulin-like growth factor-I inhibitory bioactivity in adolescent insulin-dependent diabetics Clin Endocrinol (Oxf) 1990 32 229 239 10.1111/j.1365-2265.1990.tb00859.x 1693321

[B17] Hyer SL Sharp PS Sleightholm M Burrin JM Kohner EM Progression of diabetic retinopathy and changes in serum insulin-like growth factor I (IGF I) during continuous subcutaneous insulin infusion (CSII) Horm Metab Res 1989 21 18 22 10.1055/s-2007-1009140 2925151

[B18] Schaper NC Growth hormone in type I diabetic and healthy man Acta Endocrinol (Copenh) 1990 122 Suppl 2 1 47 2110412 10.1530/acta.0.1220001

[B19] Diabetes Control and Complications Trial (DCCT) Research Group The effect of intensive treatment of diabetes on the development and progression of long-term complications in insulin-dependent diabetes mellitus N Engl J Med 1993 329 977 986 8366922 10.1056/NEJM199309303291401

[B20] Patz A Clinical and experimental studies on retinal neovascularization. XXXIX Edward Jackson Memorial Lecture Am J Ophthalmol 1982 94 715 743 10.1016/0002-9394(82)90297-5 6184997

[B21] Merimee TJ Zapf J Froesch ER Insulin-like growth factors. Studies in diabetics with and without retinopathy N Engl J Med 1983 309 527 530 10.1056/NEJM198309013090904 6348545

[B22] Wang Q Dills DG Klein R Klein BE Moss SE Does insulin-like growth factor I predict incidence and progression of diabetic retinopathy? Diabetes 1995 44 161 164 10.2337/diab.44.2.161 7859935

[B23] Hyer SL Sharp PS Brooks RA Burrin JM Kohner EM A two-year follow-up study of serum insulinlike growth factor-I in diabetics with retinopathy Metabolism 1989 38 586 589 10.1016/0026-0495(89)90222-9 2725298

[B24] Beranek M Kankova K Benes P Izakovicova-Holla L Znojil V Hajek D Vlkova E Vacha J Polymorphism R25P in the gene encoding transforming growth factor-beta (TGF-beta1) is a newly identified risk factor for proliferative diabetic retinopathy Am J Med Genet 2002 109 278 283 10.1002/ajmg.10372 11992481

[B25] Grant MB Mames RN Fitzgerald C Ellis EA Caballero S Chegini N Guy J Insulin-like growth factor I as an angiogenic agent. In vivo and in vitro studies Ann N Y Acad Sci 1993 692 230 242 10.1111/j.1749-6632.1993.tb26221.x 7692789

[B26] Danis RP Bingaman DP Insulin-like growth factor-1 retinal microangiopathy in the pig eye Ophthalmology 1997 104 1661 1669 10.1016/S0161-6420(97)30081-5 9331208

[B27] Smith LE Kopchick JJ Chen W Knapp J Kinose F Daley D Foley E Smith RG Schaeffer JM Essential role of growth hormone in ischemia-induced retinal neovascularization Science 1997 276 1706 1709 10.1126/science.276.5319.1706 9180082

[B28] Meyer-Schwickerath R Pfeiffer A Blum WF Freyberger H Klein M Losche C Rollmann R Schatz H Vitreous levels of the insulin-like growth factors I and II, and the insulin-like growth factor binding proteins 2 and 3, increase in neovascular eye disease. Studies in nondiabetic and diabetic subjects J Clin Invest 1993 92 2620 2625 10.1172/JCI116877 7504689 PMC288458

[B29] Boulton M Gregor Z McLeod D Charteris D Jarvis-Evans J Moriarty P Khaliq A Foreman D Allamby D Bardsley B Intravitreal growth factors in proliferative diabetic retinopathy: correlation with neovascular activity and glycaemic management Br J Ophthalmol 1997 81 228 233 10.1136/bjo.81.3.228 9135388 PMC1722140

[B30] Preis I Langer R Brem H Folkman J Inhibition of neovascularization by an extract derived from vitreous Am J Ophthalmol 1977 84 323 328 900228 10.1016/0002-9394(77)90672-9

[B31] Orlidge A D’Amore PA Inhibition of capillary endothelial cell growth by pericytes and smooth muscle cells J Cell Biol 1987 105 1455 1462 10.1083/jcb.105.3.1455 3654761 PMC2114828

[B32] Sato Y Rifkin DB Inhibition of endothelial cell movement by pericytes and smooth muscle cells: activation of a latent transforming growth factor-beta 1-like molecule by plasmin during co-culture J Cell Biol 1989 109 309 315 10.1083/jcb.109.1.309 2526131 PMC2115489

[B33] Antonelli-Orlidge A Saunders KB Smith SR D’Amore PA An activated form of transforming growth factor beta is produced by cocultures of endothelial cells and pericytes Proc Natl Acad Sci U S A 1989 86 4544 4548 10.1073/pnas.86.12.4544 2734305 PMC287307

[B34] Ishii DN Implication of insulin-like growth factors in the pathogenesis of diabetic neuropathy Brain Res Brain Res Rev 1995 20 47 67 10.1016/0165-0173(94)00005-A 7711767

[B35] Near SL Whalen LR Miller JA Ishii DN Insulin-like growth factor II stimulates motor nerve regeneration Proc Natl Acad Sci U S A 1992 89 11716 11720 10.1073/pnas.89.24.11716 1465388 PMC50627

[B36] Caroni P Grandes P Nerve sprouting in innervated adult skeletal muscle induced by exposure to elevated levels of insulin-like growth factors J Cell Biol 1990 110 1307 1317 10.1083/jcb.110.4.1307 2157718 PMC2116103

[B37] Crosby SR Tsigos C Anderton CD Gordon C Young RJ White A Elevated plasma insulin-like growth factor binding protein-1 levels in type 1 (insulin-dependent) diabetic patients with peripheral neuropathy Diabetologia 1992 35 868 872 10.1007/BF00399934 1383070

[B38] Jude EB Blakytny R Bulmer J Boulton AJ Ferguson MW Transforming growth factor-beta 1, 2, 3 and receptor type I and II in diabetic foot ulcers Diabet Med 2002 19 440 447 10.1046/j.1464-5491.2002.00692.x 12060054

[B39] Report of the expert committee on the diagnosis and classification of diabetes mellitus Diabetes Care 1997 20 1183 1197 9203460 10.2337/diacare.20.7.1183

[B40] Perrey C Turner SJ Pravica V Howell WM Hutchinson IV ARMS-PCR methodologies to determine IL-10, TNF-alpha, TNF-beta and TGF-beta 1 gene polymorphisms Transpl Immunol 1999 7 2 127 8 10.1016/S0966-3274(99)80030-6 10544444

[B41] Shahbazi M Fryer AA Pravica V Brogan IJ Ramsay HM Hutchinson IV Harden PN Vascular endothelial growth factor gene polymorphisms are associated with acute renal allograft rejection J Am Soc Nephrol 2002 13 260 264 11752046 10.1681/ASN.V131260

[B42] Ng DP Warram JH Krolewski AS TGF-beta 1 as a genetic susceptibility locus for advanced diabetic nephropathy in type 1 diabetes mellitus: an investigation of multiple known DNA sequence variants Am J Kidney Dis 2003 41 22 28 10.1053/ajkd.2003.50011 12500218

[B43] Merimee T The interface between diabetic retinopathy, diabetes management, and insulin-like growth factors J Clin Endocrinol Metab 1997 82 2806 2808 10.1210/jcem.82.9.4265 9284700

[B44] Russell JW Feldman EL Insulin-like growth factor-I prevents apoptosis in sympathetic neurons exposed to high glucose Horm Metab Res 1999 31 90 96 10.1055/s-2007-978704 10226787

[B45] Lundbaek K Christensen NJ Jensen VA Johansen K Olsen TS Hansen AP Orskov H Osterby R Diabetes, diabetic angiopathy, and growth hormone Lancet 1970 2 131 133 4194508 10.1016/s0140-6736(70)92706-6

[B46] Hazzalin CA Mahadevan LC MAPK-regulated transcription: a continuously variable gene switch? Nat Rev Mol Cell Biol 2002 3 30 40 10.1038/nrm715 11823796

